# Reverse Flow Superficial Sural Artery Fasciocutaneous Flap: A Comparison of Outcome between Interpolated Flap Design versus Islanded Flap Design

**DOI:** 10.29252/wjps.8.3.316

**Published:** 2019-09

**Authors:** Muhammad Saaiq, Farid Ullah Khan Zimri

**Affiliations:** Department of Plastic Surgery and Orthopedics, National Institute of Rehabilitation Medicine (NIRM), Islamabad, Pakistan

**Keywords:** Reverse flow, Superficial sural artery, Flap, Interpolated, Islanded, Necrosis

## Abstract

**BACKGROUND:**

Complex soft-tissue defects of the distal third of the leg, proximal third of foot and similar wounds around the ankle represent formidable foes for plastic surgeons. This study compared the outcome of 2-staged interpolated flap design versus single stage islanded flap design of reverse flow superficial sural artery flap.

**METHODS:**

Thirty-four patients were enrolled, while half randomly underwent interpolated flap design (group A) and for half, islanded flap design (group B). The outcome measures were frequency of epidermolysis, flap-tip necrosis, partial flap loss, total flap loss and number of secondary procedures required for addressing these complications.

**RESULTS:**

Among patients, 79.41% were male and 20.58% were females. The age range was 12-51 years (mean: 28.82±10.76 years). The wound locations were hind foot (50%), ankles (17.64%), heel (14.70%), distal third of leg (11.76%) and dorsum of proximal third of foot (5.88%). In group B, epidermolysis was noted in 35.29% of flaps, and flap tip necrosis and partial flap necrosis in 17.64%. In group A, 5.88% were tip necrosis with no other problems. In group B, 76.47% of secondary procedures were done to address various flap related complications, whereas in group A, 5.88% additional procedures were required to address the flap tip necrosis.

**CONCLUSION:**

The reverse flow superficial sural artery flap constituted a practical solution to address complex defects of the distal leg, ankle, heel and proximal foot. The 2-staged interpolated flap design considerably enhanced the flap reliability and reduced the frequency of venous congestion and resultant flap necrosis of variable proportions.

## INTRODUCTION

Complex soft-tissue defects of the distal third of the leg, proximal third of foot and similar wounds around the ankle represent formidable foes for plastic surgeons. They may be associated with exposed bones, joints, tendons, ligaments and implants. Such defects with exposed vital structures are not surmountable to cover split thickness skin grafts. There was also paucity of robust local flaps of large dimensions. Microvascular free flaps constitute viable coverage option; however, these require the availability of appropriate recipient arteries and veins for anastomosis, microsurgical expertise, microsurgical equipment and relatively prolonged operating time. Additionally, these may pose problems owing to their bulk and poor cosmesis.^1^^-^^3^

Given the aforementioned reconstructive challenges, the reverse flow superficial sural artery flap constitutes a practically suitable option for coverage. This flap depends on the retrograde flow through the median superficial sural artery from the peroneal artery through the posterolateral ankle perforators.^2^ The current study was carried out to compare the outcome of interpolated flap design versus islanded flap design of reverse flow superficial sural artery flap.

## MATERIALS AND METHODS

This comparative study was conducted at the Department of Plastic Surgery and Orthopedics, National Institute of Rehabilitation Medicine (NIRM), Islamabad, Pakistan over a period of four years, from October 1, 2014 to September 30, 2018. Informed consent was taken from the patients. The study was carried out in accordance with the Declaration of Helsinki of 1975, as revised in 2008 and anonymity of the participants was guaranteed. The study included all adult patients of either gender who presented with post-traumatic complex soft-tissue defects of the distal third of the leg, proximal third of foot, heel and similar wounds around the ankle. 

Our exclusion criteria included large size defects not manageable with reversed sural flap and injured pedicle zone. We also excluded patients with smoking, diabetes, peripheral vascular disease and varicose veins as they could confound the results of our comparative study. Initial assessment and diagnosis was made by history, physical examination and necessary investigations. The patients were hospitalized for definitive management and flap coverage. Half of the patients were randomly assigned to group A (those with interpolated flap design) and half to the group B (those with islanded flap design). 

Simple random sampling was done with computer generated random table. Maximal possible matching of the two groups was done for age, gender, and defect characteristics such as the size and location of the wound. Among all patients, the flap elevation was undertaken in prone position under tourniquet control. At the outset, the recipient defect was thoroughly debrided and wound template was taken. Flap marking was performed with drawing a straight line that joined the mid-popliteal point (i.e. central point in the midline between two heads of the gastrocnemius muscle in the popliteal fossa) to another point midway between the lateral malleolus and lateral side of the tendo-Achilles. This line marked the longitudinal vascular axis of the flap. 

The pivot point of the flap was marked 5 cm superior to the tip of the lateral malleolus. This marked the most critical and constant perforator from peroneal artery that perfuses the reverse flow flap. The flap size was marked using the defect template already taken after wound debridement. The flap was designed to be centered over the longitudinal vascular axis, pedicle length was sufficient to allow reach to the target defect and sparing the proximal one fourth (below the popliteal crease) of the posterior leg. The flap dimensions ranged from 5×5 cm^2^ to 10×15 cm^2^. 

**Fig. 1:**
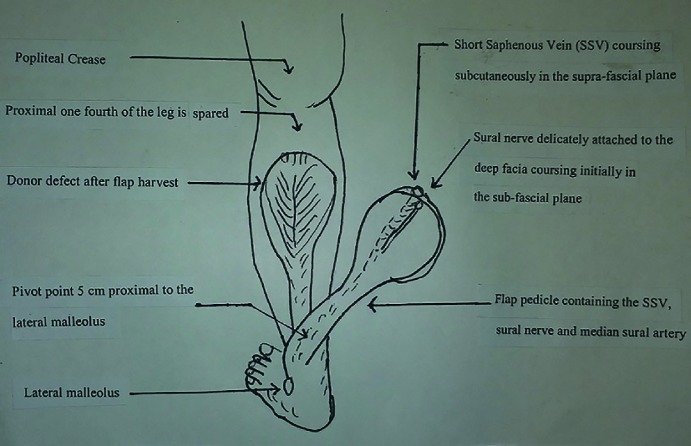
Schematic illustration of the flap on posterior aspect of left leg. The relevant surgical anatomy is elaborated

For flap elevation, an incision was made through skin and fascia along the superior border of the flap. The vascular pedicle including the median cutaneous sural nerve, short saphenous vein (SSV) and the accompanying median superficial sural artery were divided, ligated proximally and their distal continuations were included within the flap. The skin overlying the adipofascial pedicle was undermined on either side as far as the pivot point, among all islanded flaps. In case of interpolated flaps, the skin strip overlying the pedicle was harvested intact all over the length. The dissection proceeded from proximal to distal in the subfascial plane with inclusion of gastrocnemius muscle cuff around the vascular pedicle in the midline. At least 3-4 cm wide subcutaneous adipofascial pedicle was maintained to preserve its vascularity. The pedicle dissection stopped at the pivot point of the flap marked 5 cm above the lateral malleolus (Figure 1).

In case of interpolated flap design, the skin was intact over the flap pedicle, whereas in cases of islanded flap design, the skin incision was completed all around the flap leaving an intact subcutaneous adipofascial pedicle. The flap was then transposed on to the recipient defect either as an interpolated flap or through a subcutaneous tunnel. In the latter group, if pedicle compression was imminent, the skin bridge between the defect and the flap base was divided and flap transposed without a subcutaneous tunnel. The raw surface was resurfaced with split thickness skin graft (STSG). The residual flap donor site was also covered with STSG after its maximum possible primary closure. 

**Fig. 2:**
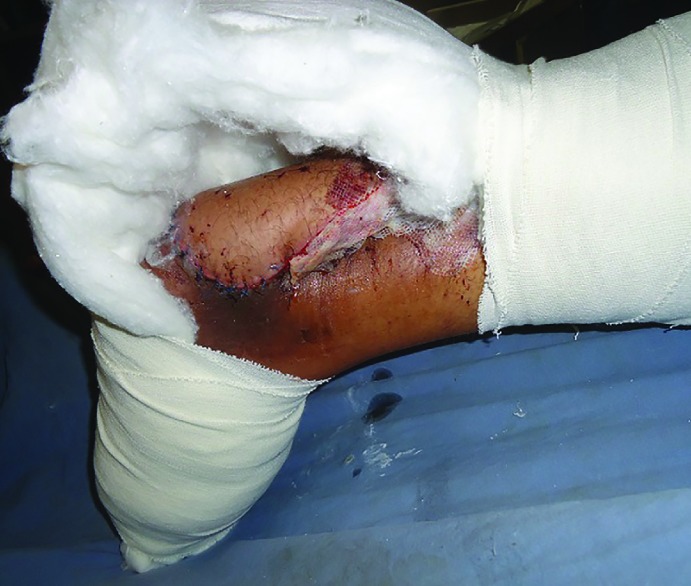
The custom made back slab of plaster of Paris was designed to protect the flap and avoid any compression or pressure on the pedicle

Postoperatively, the flap was protected with a custom made back slab of plaster of Paris designed for the foot to ensure that there was no compression or pressure on the pedicle (Figure 2). The flap was left exposed for monitoring, while rest of the wounds was covered with padded absorbent aseptic dressing. The first dressing was changed on 5^th^ postoperative day. Postoperatively, the patients were advised to lie prone so as to avoid gravitational edema of the flap. 

In case of interpolated flaps, pedicle division and insetting was undertaken after three weeks under local anesthesia. Full weight bearing was allowed by the end of 4^th^ week after first operation. The data were analyzed statistically using statistical package for social sciences (SPSS, Version 17, Chicago, IL, USA) and various descriptive statistics were employed to calculate the objectives. The outcome measures included flap survival or otherwise, frequency of complications of epidermolysis, flap-tip necrosis, partial flap loss and the number secondary procedures required for addressing these complications.

## RESULTS

There were 34 patients with 27 (79.41%) males and 7 (20.58%) females. The age ranged between 12 and 51 years (mean: 28.82±10.76 years). The flap size ranged from 5×5 cm (25 cm^2^) to 15×15 cm (225 cm^2^) with a mean size of 82.11±51.54 cm^2^. The wound locations were as follows: Hind foot (n=17; 50%), ankles (n=6; 17.64%), heel (n=5; 14.70%), distal third of leg (n=4; 11.76%) and dorsum of proximal third of foot (n=2; 5.88%). The hospital stay was 13-19 days with a mean stay of 14.61±1.93 days. In group B, the complications included epidermolysis (n=6; 35.29%), flap tip necrosis (n=3; 17.64%) and partial flap necrosis (n=3; 17.64%). In group A, we encountered one case (5.88%) of tip necrosis.

**Fig. 3:**
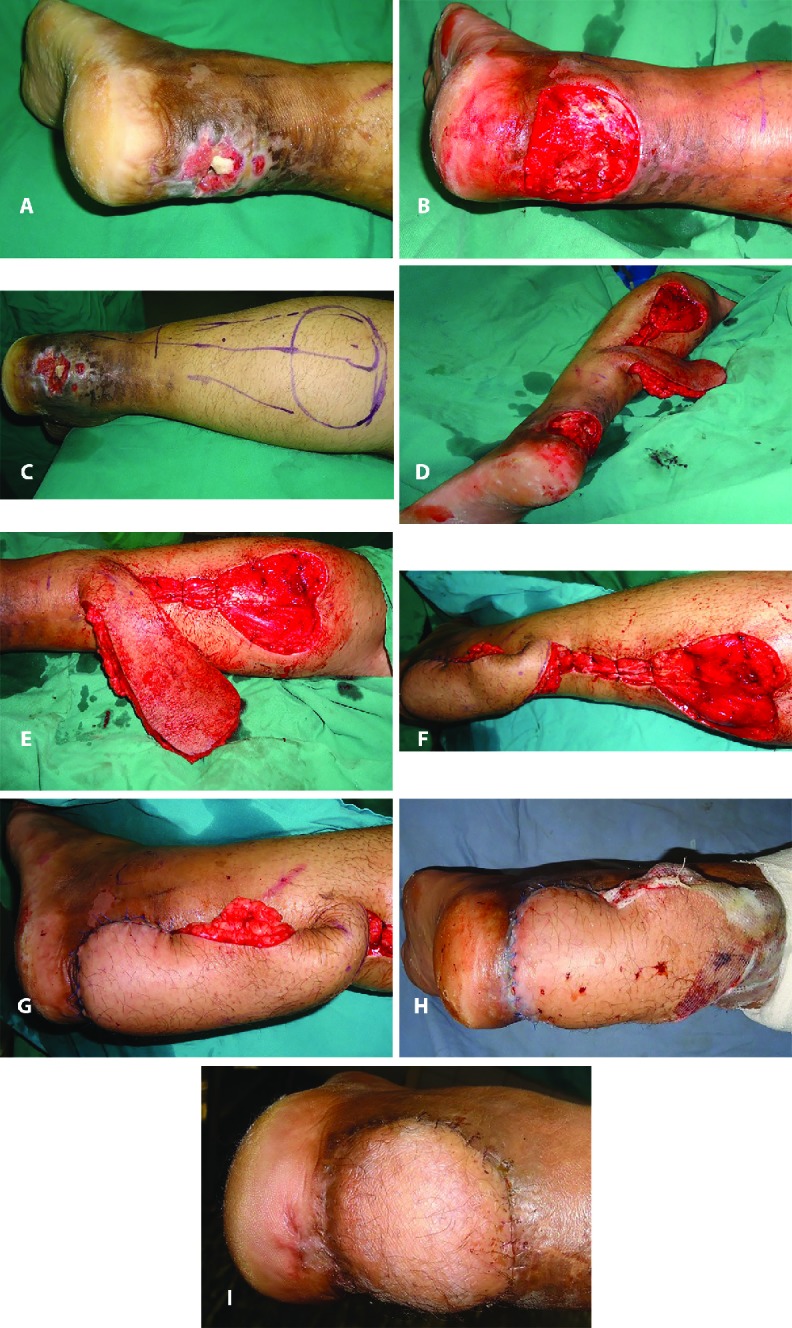
**(A):** An adult male with post-traumatic hind-foot defect of the left foot. It is of three months duration. There was exposed tendo-Achilles and unhealthy tissue around the chronic defect. **(B):** Intraoperative photograph of the same patient as in Figure 3A following thorough debridement of the wound. **(C):** Intraoperative photograph of the same patient as in Figure 3A and B showing flap markings. **(D):** Same patient as in Figure 3A, B and C showing elevated interpolated flap. **(E):** Same patient as in Figure 3A through D showing elevated flap and the target defect. **(F):** Same patient as in Figure 3A through E with interpolated flap transposed onto the defect. **(G):** Intraoperative photograph of the same patient as in Figure 3A through F, a close up view of the flap. **(H):** 5^th^ postoperative day of the same patient as in Figure 3A through G. **(I):** One month postoperative status of the same patient as in Figure 3A through G

**Fig. 4:**
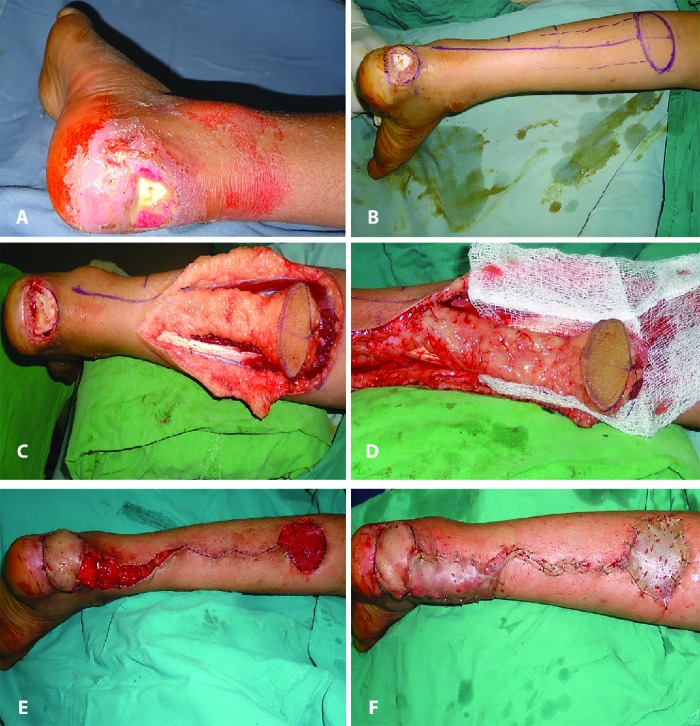
**(A):** An adult female with hind-foot defect of four weeks duration. There was exposed tendo-Achilles. **(B):** Same patient as in Figure 4A, the markings for planned islanded flap. **(C):** Same patient as in Figure 4A and B islanded flap has been elevated on the adipofascial pedicle. **(D):** A close up view of the islanded flap and its adipofascial pedicle. **(E):** Flap transposed. **(F):** Completion photograph of the same patient as in Figure 4A through E

In group B, a total of 13 (76.47%) secondary procedures were carried out to address the various flap related complications; whereas in group A, one additional procedure was required for addressing the complication of flap tip necrosis (n=1; 5.88%). Figures 3(A) through 4(F) showed two illustrative cases included in the study. The interpolated flap design was depicted in Figure 3A through Figure 3I, whereas the Islanded flap design was shown in Figure 4A through Figure 4F.

## DISCUSSION

Complex defects of the distal leg, ankle, heel and proximal foot may be caused by a variety of causes. For instance, trauma, tumor ablation, burn injury, release of contractures and pressure sore. There may be exposed bone, joint, tendons, ligaments or implants employed for fracture fixation. For the majority of such defects, the valid prudent reconstructive options were either free flaps or a reverse flow superficial sural artery flap. We chose the latter option owing to the fact that it is easier, quicker and does not require microsurgery in addition to providing less bulky and pliable soft tissue coverage.^2^^,^^4^^,^^5^

The reverse flow superficial sural artery flap has a robust and reliable vascular perfusion at the ankle. At this level, it is perfused by the following known sources of blood supply: (i) septocutaneous perforators arising from the peroneal artery, (ii) fasciocutaneous perforators from the peroneal artery, (iii) neurocutaneous perforators from the sural nerve, (iv) venocutaneous perforators from the SSV, and (v) fasciocutaneous and septocutaneous perforators from the posterior tibial artery.^2^^,^^4^^,^^5^

Among the aforementioned sources, the most consistent ones are the septocutaneous perforators from the peroneal artery. These are 3-6 in number on each side, with the most distal being located 4-7 cm proximal to the lateral malleolus. These perforators directly join the superficial sural arteries.^2^^,^^4^^,^^5^ In our patients, we routinely employed the gastrocnemius muscle cuff technique, while harvesting the flap in the popliteal fossa. In the midline, a generous cuff of muscle was harvested around the sural pedicle. This helped to ensure preservation of the delicate vascular plexus in the most precarious leading part of the flap, hence increasing the viability of the flap tip.^2^^,^^6^

At the proximal aspect of the flap below the popliteal crease, once the incision was made, the SSV lied subcutaneously in the suprafacial plane; whereas the sural nerve lied in the subfacial plane being delicately attached to the deep surface of the deep fascia. Here, if the dissection was careless; it was easy to damage the delicate vascular plexus of the perineural origin. Hence, the inclusion of muscle cuff safeguards against any inadvertent damage to the critical vascularity in this most critical part of the flap. We recommend harvesting the sural nerve along with its surrounding adipofascial tissue attaching the nerve to the deep fascia. 

In none of our patients, we kept the pivot point to be distal to the standard 5 cm proximal to the tip of lateral malleous. Where possible, we maintained this point even higher in order to incorporate the potential perforator lying higher. We did not perform Doppler mapping in our patients. Some published studies have reported on more distalization of the pivot point. In our study, we excluded patients with known co-morbidities such as smoking, diabetes, peripheral vascular disease and varicose veins as they could confound the results of our comparative study.

Several published studies have reported higher risk of flap necrosis among these patients compared to those without these conditions. The risk factors of venous insufficiency, arteriosclerosis and diabetes constitute the ‘unhappy triad’ in this regard. Among all these high risk patients, flap delay would be the key to maximizing flap reliability. Also, flap delay should be considered in the presence of obesity and extended flap design.^2^^,^^7^^-^^9^ Impaired venous outflow represents the most challenging aspect of the reverse flow superficial sural artery flap.^2^^,^^10^^,^^11^

When we elevated and transposed the flap, the SSV got oriented in a reverse direction. As the vein had competent valves, venous outflow was not allowed in the reverse direction. An additional venous outflow mechanism was thought to be provided by the venules that accompanied the vasa nervorum of the sural nerve and vasa vasorum of the SSV. This system ensured an antegrade flow towards the distal leg perforators and eventually into the vena comitantes of the peroneal artery. Venous blood can flow from SSV to these concomitant veins via oscillating avalvular veins, thus passing blood from superficial to deep venous systems. It is imperative to safeguard these systems during flap elevation.^2^^,^^10^^,^^11^

In the past, a variety of techniques have been tried to improve the venous drainage of the flap. For instance, supercharging the flap (by anatomizing the SSV to any recipient area vein), exteriorizing the divided proximal stump of the SSV, intermittent phlebotomy, use of medicinal leeches and use of low molecular weight Dextran.^2^^,^^12^^-^^14^ In our series, we found the interpolated flap to be superior to the islanded design with respect to the safety and reliability of the flap in terms of reducing the complications of epidermolysis, flap-tip necrosis, partial flap loss, total flap necrosis and the number of secondary procedures required for addressing these complications. Our findings conform to those of various published studies.^15^^-^^18^

Vendramin *et al.*^19^ from Brazil devised a new technique of leaving intact at least 1.5 cm strip of skin over the adipofascial pedicle in addition to recruiting at least 1 cm extra fasciosubcutaneous margin underneath the skin portion of the flap. With this technical modification, the author found remarkable reduction in incidence of partial necrosis, whereas no cases of total flap necrosis were visible. Dhamangaonkar *et al.*^20^ from India employed the flap as a single stage procedure with an intact skin all over the adipofascial pedicle and delivered the flap to the target defect through an open passage created by incising the skin bridge between the donor and recipient sites.^20^

In this way, not only tunneling was avoided but also the broad intact skin was provided for the venous drainage of the reverse flap. There was 89.21 % rate of uneventful healing of the flap with edge necrosis encountered among only 8.25% of flaps.^20^ Herlin *et al.*^21^ from France improvised the traditional technique by harvesting a skin blade (1.5-2 cm wide) over the pedicle (hence a fasciocutaneous pedicle), making a back-cut until the defect and suturing the flap to the edges alongside. They noticed remarkable reduction in the incidence of distal venous congestion of the flaps.

In majority of the published studies, the reversed sural flap has been used in an islanded fashion. Variable complication rates and various risk factors have been highlighted by the authors in an attempt to provide possible explanation for the given complications. Venous congestion through the valved SSV system has been reported to be the most frequent root cause of the flap related complications.^2^^,^^4^^,^^22^ Quirino *et al.*^23^ reported epidermolysis to be the most frequent complication encountered among 36% of their flaps. 

Almeida *et al.*^24^ reported partial flap necrosis among 22% of cases, whereas total flap necrosis in 4.2% and venous congestion amongst 4.1%. Wei *et al.*^25^ in their retrospective review of 179 sural flaps, reported uneventful flap survival among 78.77% of cases, whereas various complications amongst 21.22% of flaps. Partial flap necrosis was encountered among 11.17%, distal de-epithelialization among 6.70% and wound dehiscence among 3.35% of cases. The authors found that partial necrosis was more likely, when top-edge of the flap was located in the upper 1/9 of the calf, when length width ratio of the flap was ≥5:1 or when width was ≥8 cm.

Unresolved mysteries still continue to surround the exact arterial perfusion as well as venous outflow of the reverse flow superficial sural artery flap. The search for their detailed understanding as well as relevant solutions should be continued. We suggest future multicentre prospective studies to address these knowledge gaps. The reverse flow superficial sural artery flap constitutes a practical solution to address complex defects of the distal leg, ankle, heel and proximal foot. The 2-satged interpolated flap design considerably enhanced the flap reliability and reduced the frequency of venous congestion and resultant flap necrosis of variable proportions.

## CONFLICT OF INTEREST

The authors declare no conflict of interest.
